# Tobacco-attributable burden of alzheimer’s disease and other dementias in China and ASEAN: Temporal trends and projections – an observational study based on GBD 2021

**DOI:** 10.1097/MD.0000000000050974

**Published:** 2026-10-09

**Authors:** Piaoyun Wen, Wenwen Liu, Yanjiang Li, Mengmeng Liao, Haofeng Qin, Jiaxi Ye, Ziyong Deng, Shuqi Cui, Yongli Zhao, Xiaowen Wang, Yingzi Huang, Huamin Tang, Hui Qin, Kaihua Wang, Qijing Qin

**Affiliations:** a Faculty of Zhuang Clinical Medicine of Guangxi University of Chinese Medicine, Nanning, Guangxi, China; b International Zhuang Medicine Hospital Affiliated to Guangxi University of Chinese Medicine, Nanning, Guangxi, China.

**Keywords:** Alzheimer’s disease and other dementias, ASEAN countries, China, disease burden, Time-series forecasting

## Abstract

This study aimed to systematically analyze the disease burden of Alzheimer’s disease and other dementias (ADOD) attributed to tobacco in China and ASEAN countries from 1990 to 2021, and predict the disease burden in the next ten years to characterize the disparities in disease burden between the 2 sides, and to conduct a preliminary exploration of factors that may be associated with these disparities. Data on ADOD deaths, mortality, DALYs and DALY rates attributed to tobacco in China and ASEAN countries were extracted from GBD 2021, and the trend of disease burden was analyzed using Joinpoint regression model. R software was used for data analysis and visualization, and Joinpoint regression model was used to calculate the APC of each indicator; the disease burden attributable to tobacco in China and ASEAN countries was analyzed through the SDI; and the ARIMA was used to predict ADOD from 2022 to 2035. In China, the number of ADOD deaths (1990: 8126.47, 95% CI: 1822.19–22,577.84), mortality rate, DALYs, and DALY rate attributable to tobacco all exhibited significant and sustained upward trends from 1990 to 2021, and consistently exceeded the corresponding levels in the other ten ASEAN countries. The APC for mortality and DALY rates showed a significant increase in China and most ASEAN countries, with particularly rapid increases in mortality in China, Thailand, Cambodia, and Indonesia. Correspondingly, the DALY rate rose in most countries, indicating a growing overall disease burden. An exception was Myanmar, where the burden decreased. The burden of tobacco-attributable ADOD was consistently higher among males than females in all countries. Analysis based on SDI revealed a lower and more stable disease burden in high-SDI areas, whereas medium- and low-SDI countries demonstrated an upward trend. ARIMA model projections for 2022–2035 suggest that while mortality trends are diverse, an increasing trend is anticipated in most countries. The disease burden of ADOD attributable to tobacco use has continued to rise in China and ASEAN countries. This trend suggests a potential role for tobacco control in the prevention of ADOD, and also points to the value of strengthening tobacco regulation and paying early attention to cognitive interventions.

## 1. Introduction

Alzheimer’s Disease and Other Dementias (ADOD) represent a highly heterogeneous group of neurodegenerative diseases, including Alzheimer’s disease, vascular dementia, dementia with Lewy bodies, and other types. Despite differences in etiology and clinical presentation, they share core features: progressive cognitive decline, behavioral abnormalities, and loss of functional independence. According to the World Health Organization, ADOD was the seventh leading cause of death globally in 2021, following ischemic heart disease, stroke, chronic obstructive pulmonary disease, and lower respiratory tract infections.^[1]^ With population aging intensifying, the proportion of ADOD in total deaths continues to rise. The global number of affected individuals reached 51.6 million, and exceeded 55 million in 2023.^[2,3]^

Tobacco exposure is a major modifiable risk factor for chronic diseases. It increases ADOD risk through mechanisms such as vascular endothelial injury, oxidative stress, and neuroinflammation.^[4,5]^ Smoking promotes vascular pathology—a well-established dementia risk factor—and exposes the brain to nicotine and other toxins that exacerbate oxidative stress and inflammation, both implicated in ADOD pathogenesis.^[6]^

Geographically proximate and culturally interconnected, China and the ASEAN region face dual public health challenges: widespread tobacco use and an aging population.^[7]^ Currently, systematic comparisons and dynamic trend analyses of the burden of ADOD attributable to tobacco exposure in China and ASEAN countries remain limited. This study, based on the GBD 2021 database, aims to systematically compare the burden of ADOD attributable to tobacco exposure in China and ASEAN countries from 1990 to 2021, including its level, temporal trends, and differences across countries, sexes, and sociodemographic index (SDI) strata, as well as project mortality changes in China and ASEAN countries up to 2035. Joinpoint regression analysis is used to identify trend turning points and calculate annual percentage changes to test the statistical significance of downward or upward trends across countries. Meanwhile, an ARIMA model is constructed using R language to conduct short-term projections of the burden changes from 2022 to 2035. Through the systematic comparison described above, this study helps to elucidate the distribution characteristics and evolution patterns of regional disease burden.

## 2. Materials and methods

### 2.1. Data sources

This study utilized data from the GBD 2021 database. The GBD 2021 systematically collates data on prevalence, incidence, and disability-adjusted life years (DALYs) for 288 causes of death, 371 diseases and injuries, and 88 risk factors across 204 countries and regions from 1990 to 2021, including subnational estimates for 21 countries and regions.^[8]^ The Socio-demographic Index (SDI) was employed as a composite indicator of societal development level. Ranging from 0 to 1, the SDI is derived from a country’s average educational attainment, economic status, and total fertility rate; a higher SDI value indicates a higher level of societal development.^[9]^ The exposure factor in this study is “tobacco exposure,” defined in accordance with the Global Burden of Disease Study 2021 (GBD 2021), which includes active smoking, secondhand smoke exposure, and smokeless tobacco use. For ease of expression, the term “tobacco” in this paper refers collectively to these 3 forms of exposure. The level of exposure was quantified using the population-attributable fraction (PAF) provided by the GBD. The outcome variable is the burden of ADOD attributable to tobacco, with the specific scope of ADOD based on the GBD 2021 classification, including Alzheimer’s disease and other types of dementia (e.g., vascular dementia). Disease burden is expressed as age-standardized mortality and disability-adjusted life year (DALY) rates. It is important to note that the GBD estimates are model-based projections rather than directly observed surveillance data. These definitions are fully consistent with the GBD 2021 standards to ensure the reproducibility and international comparability of the study. The GBD database has already stratified and adjusted for major confounders, including age, sex, and the Socio-demographic Index (SDI). Due to data limitations, this study did not control for individual-level confounders (e.g., education, lifestyle) or potential biases (e.g., information bias), and these limitations are addressed in the discussion section. Furthermore, all estimates from GBD 2021 are presented with their 95% uncertainty intervals (UIs), which represent the 2.5th and 97.5th percentiles of 1000 draws from the posterior distribution of the respective quantities.

### 2.2. Statistical analysis

Between-group comparisons were performed using descriptive statistics and 95% uncertainty intervals (UIs). Given the inherent uncertainty in GBD estimates, this study primarily focused on the practical differences in point estimates and interpreted the results in the context of the UI ranges. Practical significance was determined based on the magnitude of fold‑differences (e.g., a ≥ 2‑fold difference was considered practically meaningful, a ≥ 2‑fold difference was prespecified as a conservative threshold to minimize overinterpretation of minor fluctuations inherent in model‑based estimates), rather than relying solely on the overlap of UIs.

## 3. Method of processing data

### 3.1. Joinpoint regression model

Data were collected from the GBD 2021 database and organized using Microsoft Excel 2024. A log-linear regression model was then established using Joinpoint regression software. The rate of change was calculated as follows: [(Value in 2021 − Value in 1990)/ Value in 1990] × 100%. The Joinpoint model employs the least squares method to estimate the changing pattern of disease incidence, avoiding the subjectivity inherent in typical linear trend analyses.^[10]^ Data analysis and visualization were performed using R 4.5.1. The annual percent change (APC) and its 95% confidence interval (CI) for each indicator were calculated using the Joinpoint regression model to analyze the changes in the ADOD disease burden attributable to tobacco in China and ASEAN countries. An increasing trend was indicated when the APC and the lower bound of its 95% CI were greater than zero, whereas a decreasing trend was determined when the upper bound was less than zero.^[11]^ A two-sided significance level of α = 0.05 was applied.

### 3.2. Autoregressive moving average (ARIMA) prediction model

The ARIMA model consists of 3 components: autoregressive (AR), integrated (I), and moving average (MA), which are used to capture the autocorrelation of time series, eliminate non-stationarity, and smooth random fluctuations, respectively.^[12,13]^ The model expression is denoted as ARIMA (p, d, q), where p represents the autoregressive order, d indicates the degree of differencing, and q specifies the moving average order.^[12]^ In contrast to exponential smoothing or machine learning approaches, ARIMA is better suited to address the non-stationarity inherent in the mortality series of this study and provides more stable parameter estimates and stronger interpretability when extrapolating from small samples. Using R version 4.5.1, the analysis was conducted in a stepwise manner. First, the data were transformed into a time series object, and the stationarity of the series was examined using the ndiffs function to determine the required order of differencing. Subsequently, the auto.arima function was employed to automatically fit the optimal model. Model validity was assessed through residual diagnostics, including the Ljung–Box test and Q–Q plot. Model fit accuracy was evaluated using RMSE and MAPE, and model validity was assessed using the Ljung–Box test (lag = 10), where *P* > .05 indicates no significant residual autocorrelation; all statistics were obtained via the summary(fit) function in R’s forecast package. The GBD 2021 study provides continuous annual estimates from 1990 to 2021 with no missing years. The forecasting horizon was set to 2035 (14 years beyond 2021) to align with WHO’s long-term strategy and ensure comparability with prior GBD-based studies. Notably, the forecasts reflect historical trends only up to 2021. Finally, mortality rates were projected for 2022 to 2035, with visualizations showing historical trends, predicted means, and 95% confidence intervals.

### 3.3. Ethics approval and consent to participate

Ethical approval was waived for this study because it utilizes exclusively aggregated, de-identified data from the Global Burden of Disease Study 2021 (GBD 2021). The GBD study itself is compliant with all relevant ethical regulations, and its data collection procedures were approved by the Institutional Review Board of the University of Washington.

## 4. Results

### 4.1. ADOD disease burden attributable to tobacco

Between 1990 and 2021, the burden of disease attributable to tobacco in China and several ASEAN countries showed a marked upward trend. In China, the number of deaths increased from 8126.50 (95% UI: 1822.19–22,577.84) to 24,897.30 (95% UI: 5866.80–70,648.4), and DALYs rose from approximately 214,824.1 person-years to 602,501.1 person-years, far exceeding the levels observed in ASEAN countries. Among ASEAN countries, Indonesia, Thailand, and Vietnam experienced the most prominent increases. In Indonesia, deaths exceeded one thousand (1155.9 cases), and DALYs rose to 32,681.90 person-years. In Thailand and Vietnam, deaths approached or exceeded 600 cases, with DALYs exceeding 15,000 person-years in both countries. In terms of rate indicators, the mortality rate in China increased from 0.69 to 1.75 per 100,000 population, and the DALY rate rose from 18.26 to 42.35 per 100,000 population. An even more pronounced increase was observed in Thailand, where the mortality rate rose from 0.44 to 1.11 per 100,000, and the DALY rate increased from 10.83 to 23.43 per 100,000. By contrast, Brunei and Singapore had lower baseline rates and experienced only modest increases. In Myanmar, the DALY rate decreased from 12.44 to 10.50 per 100,000, albeit with wide uncertainty intervals. In 2021, the number of ADOD deaths attributable to tobacco in China was 24,900, which is 5 times the total of approximately 5000 deaths across all ten ASEAN countries. The DALY rate in China (42.35 per 100,000) was approximately 3.4 times the ASEAN average (12.5 per 100,000). Overall, China exhibited the most substantial increase in disease burden, while Thailand recorded the largest rise in rate indicators among ASEAN countries (Table 1, Fig. 1A–D).

**Table 1:** Tobacco-attributed alzheimer’s disease and other dementias burden in China and ASEAN countries (1990–2021)

Country/Pr oject	1990 (95% UI)			2021 (95% UI)			Percentage Change (%)		
	Male	Female	Total	Male	Female	Total	Male	Female	Total
**Number of deaths(n**)									
China	5911.76 (1321.81~16753.81)	2214.72 (474.64~5921.82)	8126.47 (1822.19~22577. 84)	19818.60 (4746.14~58423. 39)	5078.71 (1056.17~14243. 84)	24897.31 (5866.8~70648.4)	235.24	129.32	206.37
ASEAN	1121.09	340.13	1461.23	2809.14	663.13	3472.29	150.57	94.96	137.63
Brunei	0.5 (0.10~1.49)	0.2 (0.04~0.67)	0.69 (0.16~2.12)	0.78 (0.17~2.33)	0.36 (0.08~1.03)	1.14 (0.24~3.41)	56.00	80.00	65.22
Cambodia	24.50 (5.64~71.03)	4.73 (0.99~13.74)	29.23 (6.71~86.20)	80.32 (17.72~228.51)	16.14 (3.32~48.89)	96.46 (21.73~276.24)	227.84	241.23	230.00
Indonesia	331.72 (71.07~984.49)	55.65 (11.47~159.68)	387.38 (83.56~1147.78)	980.46 (228.20~2934.3 3)	175.40 (38.20~502.34)	1155.87 (276.47~3446.9 9)	195.57	215.18	198.38
Laos	7.77 (1.72~22.34)	1.56 (0.31~4.88)	9.33 (2.06~27.08)	21.81 (5.14~61.66)	4.34 (0.95~12.04)	26.15 (6.40~73.13)	180.69	178.20	180.28
Malaysia	66.42 (14.89~188.80)	14.2 (2.75~44.11)	80.62 (18.48~134.11)	134.63 (29.95~391.24)	21.76 (4.86~63.45)	156.39 (35.35~455.48)	102.69	53.24	93.98
Myanmar	101.16 (21.86~30 7.76)	68.9 (13.89~196.6 0)	170.06 (38.40~48 3.65)	153.73 (33.27~46 7.17)	86.12 (17.82~261. 43)	239.85 (53.22~72 9.09)	51.97	24.99	41.04
Philippines	132.57 (29.50~38 0.93)	73.68 (15.70~216. 10)	206.25 (46.68~60 0.10)	276.44 (61.70~83 2.25)	152.49 (33.46~45 8.33)	428.93 (97.99~12 74.47)	108.52	106.96	107.97
Singapore	4.00 (0.86~11.72)	3.04 (0.64~9.02)	7.04 (1.60~20.60)	13.89 (3.01~37.66)	9.32 (1.92~24.61)	23.22 (5.05~61.26)	247.25	206.58	229.83
Thailand	193.47 (41.34~552.61)	54.09 (12.55~146.17)	247.57 (52.98~693.45)	616.7 (141.92~1747.0 0)	126.36 (26.16·349.86)	743.06 (170.63~2054.7 1)	218.75	133.61	200.14
Vietnam	258.98 (56.30~70 8.95)	64.08 (15.26~183. 71)	323.06 (72.66~90 2.01)	530.38 (117.16~1 492.36)	70.84 (15.09~195. 24)	601.22 (132.85~1 640.65)	104.79	10.55	86.1
**Mortality rate (per 100,000 population**)									
China	0.97 (0.22~2.76)	0.39 (0.08~1.04)	0.69 (0.15~1.92)	2.72 (0.65~8.02)	0.73 (0.15~2.05)	1.75 (0.41~4.97)	180.41	87.18	153.62
ASEAN	0.50	0.17	0.33	0.79	0.21	0.5	58	23.53	51.52
Brunei	0.36 (0.08~1.08)	0.16 (0.03~0.55)	0.27 (0.06~0.82)	0.33 (0.07~0.98)	0.17 (0.04~0.49)	0.25 (0.05~0.76)	−8.33	6.25	−7.4
Cambodia	0.5 (0.12~1.46)	0.09 (0.02~0.25)	0.28 (0.07~0.84)	0.96 (0.21~2.73)	0.19 (0.04~0.56)	0.57 (0.13~1.62)	92.00	111.11	103.57
Indonesia	0.36 (0.08~~1.07)	0.06 (0.01~0.17)	0.21 (0.05~0.62)	0.69 (0.06~2.08)	0.13 (0.03~0.36)	0.41 (0.10~1.24)	91.67	116.67	95.24
Laos	0.38 (0.08~1.09)	0.07 (0.01~0.23)	0.22 (0.05~0.65)	0.59 (0.14~1.66)	0.12 (0.03~0.33)	0.35 (0.09~0.99)	55.26	71.43	59.09
Malaysia	0.74 (0.17~2.12)	0.16 (0.03~0.50)	0.46 (0.10~1.33)	0.82 (0.18~2.38)	0.14 (0.03~0.41)	0.49 (0.11~1.43)	10.81	-12.5	6.52
Myanmar	0.51 (0.11~1.54)	0.34 (0.07~0.96)	0.42 (0.09~1.20)	0.56 (0.12~1.72)	0.3 (0.06~0.89)	0.43 (0.09~1.29)	9.8	-11.76	2.38
Philippines	0.42 (0.09~1.20)	0.24 (0.05~0.69)	0.33 (0.07~0.95)	0.48 (0.11~1.15)	0.27 (0.06~0.82)	0.38 (0.09~1.13)	14.28	12.5	15.15
Singapore	0.26 (0.06~0.76)	0.2 (0.04~0.60)	0.23 (0.05~0.68)	0.48 (0.10~1.29)	0.33 (0.07~0.87)	0.41 (0.09~1.07)	84.61	65	78.26
Thailand	0.69 (0.15~1.96)	0.19 (0.04~0.51)	0.44 (0.09~1.22)	1.92 (0.44~5.43)	0.37 (0.08~1.01)	1.11 (0.26~3.08)	178.26	94.73	152.27
Vietnam	0.78 (0.17~2.14)	0.18 (0.04~0.52)	0.47 (0.11~1.32)	1.06 (0.23~2.98)	0.14 (0.03~0.39)	0.60 (0.13~1.64)	35.89	-22.22	27.66
**DALYs(n**)									
China	166768.77 (67785.75~38939 5.30)	48055.35 (19076.62~10647 6.99)	214824.12 (22552.25~49816 8.63)	497165.5 (208749. 72~1158493.86)	105335.58 (42541.44~23966 3.50)	602501.10 (257945.38~1379 582.96)	198.12	119.20	180.46
ASEAN	31155.42	8519.33	39674.74	73216.58	14763.58	87980.12	135.00	73.3	121.75
Brunei	13.15 (5.80~31.23)	4.66 (1.88~12.10)	17.81 (7.70~44.02)	23.77 (10.21~56.35)	9.49 (3.82~21.69)	33.27 (14.11~77.69)	80.76	103.65	86.80
Cambodia	674.43 (286.10~1572.2 1	140.90 (56.83~323.32)	815.33 (348.30~1890.4 4)	2029 (833.11~4785.1 7)	427.24 (168.19~1071.8 7)	2456.24 (1004.86~5893. 47) )	200.85	203.22	201.26
Indonesia	10060.28 (4616.74~23186.47)	1476.66 (609.18~3327.1 36)	11536.94 (5301.60~26170.21)	28616.38 (12345.38~6813 0.05)	4065.52 (1632.43~9817. 30)	32681.9 (13974.34~7998 8.05)	184.45	175.32	183.28
Laos	248.56 (109.29~559.68)	45.9 (19.26~109.36)	294.46 (128.24~657.68)	584.60 (254.33~1360.8 7)	110.83 (41.95~250.40)	695.43 (299.41~1605.3 4)	135.19	141.46	136.37
Malaysia	1460.54 (321.27~3426.9 7)	264.16 (94.73~651.79)	1724.7 (723.37~3927.7 5)	3097.74 (1328.48~7629. 10)	435.53 (167.16~1048.5 2)	3533.27 (1523.02~8417. 42)	112.09	64.87	104.86
Myanmar	3049.61 (1418.82~6647.9 6)	1981.84 (853.51~4321.32 4)	5031.45 (2304.91~11010. 88)	3818.92 (1614.37 ~8950.76)	2104.87 (838.78~5008.04)	5923.78 (2466.02 ~14355.87)	25.23	6.21	17.73
Philippines	3957.12 (1767.87~8763.3 6)	1944.49 (831.68~4288.93)	5901.6 (2677.77~12870. 95)	7805.01 (3337.38~18358. 53)	3434.33 (1466.30~8128.6 5)	11239.34 (4918.55~26001. 47)	97.24	76.62	90.44
Singapore	123.96 (52.07~28 1.87)	61.44 (22.35~148. 52)	185.39 (76.36~426.34)	396.69 (180.12~836.97)	168.15 (62.45~372.32)	564.84 (246.84~1180.8 7)	220.01	173.68	204.68
Thailand	4893.39 (1994.76 ~11211.16)	1254.34 (519.85~ 2948.20)	6147.74 (519.85~ 2948.20)	13112.05 (5395.88 ~29777.46)	2509.91 (949.83~ 5372.91)	15621.96 (6409.23 ~34864.13)	167.95	100.1	154.11
Vietnam	6674.38 (2759.46 ~15306.27)	1344.94 (543.21~ 3078.51)	8019.32 (3341.27 ~18248.85)	13732.42 (5525.17 ~32578.16)	1497.71 (555.36~ 3359.40)	15230.13 (6108.95 ~36090..72)	105.75	11.36	89.92
**DALY rate(per 100,000 population**)									
China	27.48 (11.17~64.16)	8.44 (3.35~18.69)	18.26 (7.53~42.34)	68.28 (28.67~159.11)	15.16 (6.12~34.50)	42.35 (18.13~96.97)	148.47	79.62	131.92
ASEAN	13.62	4.14	8.83	19.85	4.78	12.29	45.74	15.46	39.18
Brunei	9.57 (4.22~22.72)	3.83 (1.54~9.93)	6.87 (16.99~2.97)	9.99 (4.29~23.67)	4.46 (1.79~10.18)	7.37 (3.13~17.22)	4.39	16.45	7.28
Cambodia	13.83 (5.87~32.25)	2.61 (1.05~5.99)	7.94 (3.39~18.40)	24.25 (9.96~57.18)	4.92 (1.94~12.35)	14.41 (5.90~34.58)	75.34	88.5	81.49
Indonesia	10.9 (5.00~25.12)	1.59 (0.66~3.59)	6.24 (2.87~14.15)	20.27 (8.74~48.25)	2.95 (1.19~7.13)	11.72 (5.01~28.68)	85.96	85.53	87.82
Laos	12.14 (5.34~27.34)	2.16 (0.91~5.15)	7.06 (3.08~15.77)	15.75 (6.85~36.67)	3.02 (1.14~6.83)	9.43 (4.06~21.76)	29.74	39.81	33.57
Malaysia	16.38 (6.96~38.44)	3.02 (1.08~7.45)	9.76 (4.09~22.23)	18.82 (8.07~46.35)	2.84 (1.09~6.83)	11.11 (4.79~26.46)	14.9	-5.96	13.83
Myanmar	15.3 (7.12~33.36)	9.66 (4.16~21.07)	12.44 (5.70~27.23)	14.02 (5.93~32.87)	7.21 (2.87~17.16)	10.5 (4.37~25.44)	-8.37	-25.36	-15.59
Philippines	12.47 (5.57~27.61)	6.22 (2.66~13.72)	9.37 (4.25~20.43)	13.59 (5.81~31.97)	6.15 (2.62~14.55)	9.92 (4.34~22.96)	8.98	-1.25	5.87
Singapore	8.08 (3.39~18.37)	4.06 (1.48~9.82)	6.08 (2.51~13.99)	13.61 (6.18~28.72)	5.98 (2.22~13.23)	9.86 (4.31~20.62)	68.44	47.29	62.17
Thailand	17.39 (7.09~39.83)	4.38 (1.82~10.30)	10.83 (4.46~24.59)	40.73 (16.76~92.5 1)	7.28 (2.75~15.58)	23.43 (9.61~52.28)	134.21	66.21	116.34
Vietnam	20.15 (8.33~46.20)	3.83 (1.55~8.77)	11.75 (4 90~26 75)	27.46 (11 04~65 14)	2.98 (1 11~6 68)	15.19 (6 09~35 99)	36.28	−22.19	29.28

Percentage change = (2021 value − 1990 value)/ 1990 value × 100% (positive = increase, negative = decrease). Values were calculated separately for males.

ASEAN Total is the sum of deaths across ASEAN countries. 95% UI not calculable.

**Figure 1.**
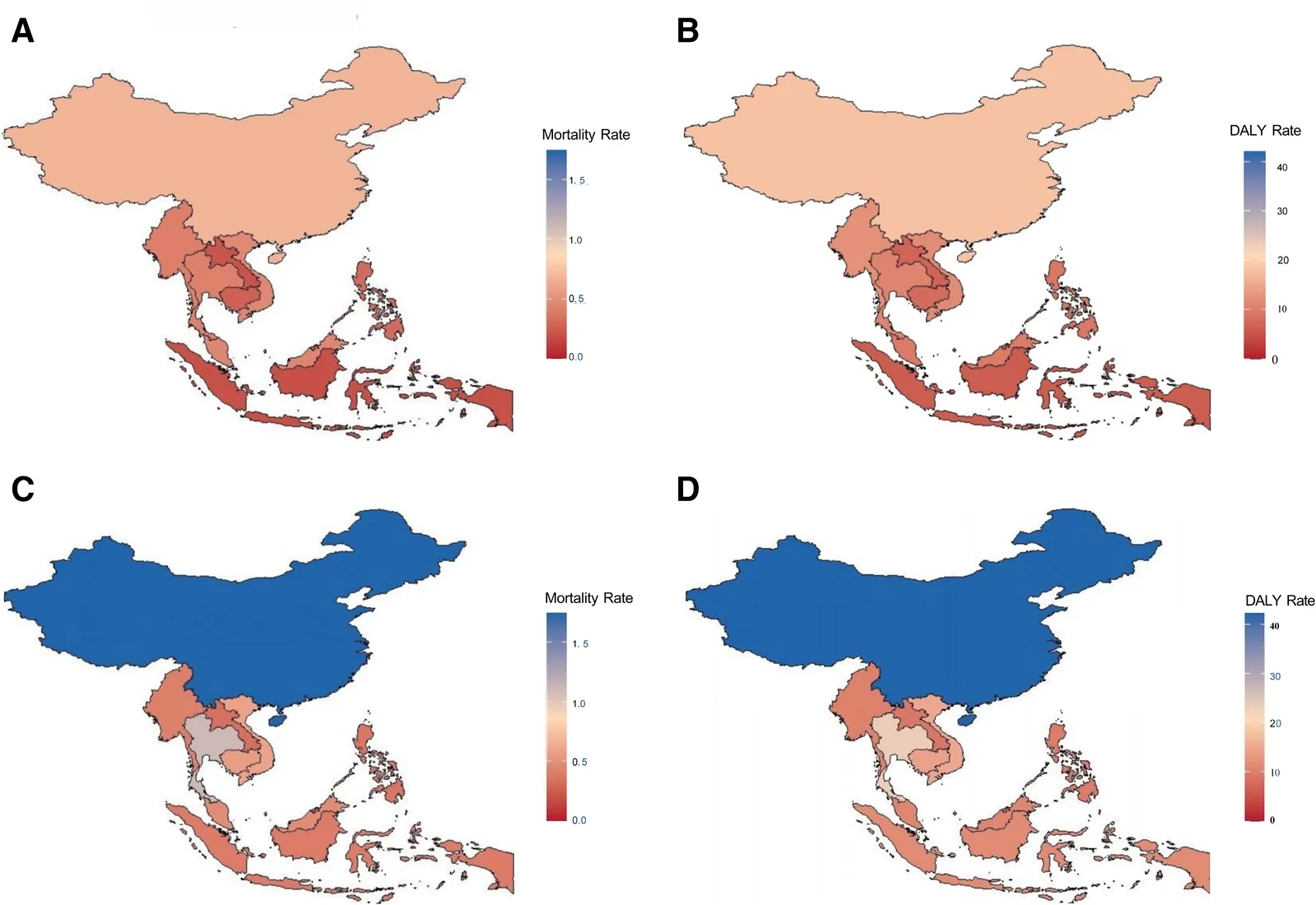
(A) Distribution of ADOD mortality rate attributable to tobacco in China and ASEAN countries in 1990; (B) Distribution of ADOD DALY rate attributable to tobacco in the same regions in 1990; (C) Distribution of ADOD mortality rate attributable to tobacco in 2021; (D) Distribution of ADOD DALY rate attributable to tobacco in 2021. The color gradient from red to blue indicates increasing rates (red representing lower values, blue representing higher values). ADOD = Alzheimer’s disease and other dementias; DALY = disability-adjusted life year.

Between 1990 and 2021, the overall mortality rate and DALY rate of ADOD attributable to tobacco showed an increasing trend in both China and ASEAN countries. China and Thailand exhibited the most pronounced increases, particularly after 2010. In contrast, Brunei, Malaysia, Myanmar, and Vietnam experienced an initial decline followed by a rise in both mortality rate

and DALY rates between 2000 and 2015, with relatively moderate trend shifts (Fig. 2A, B).

**Figure 2.**
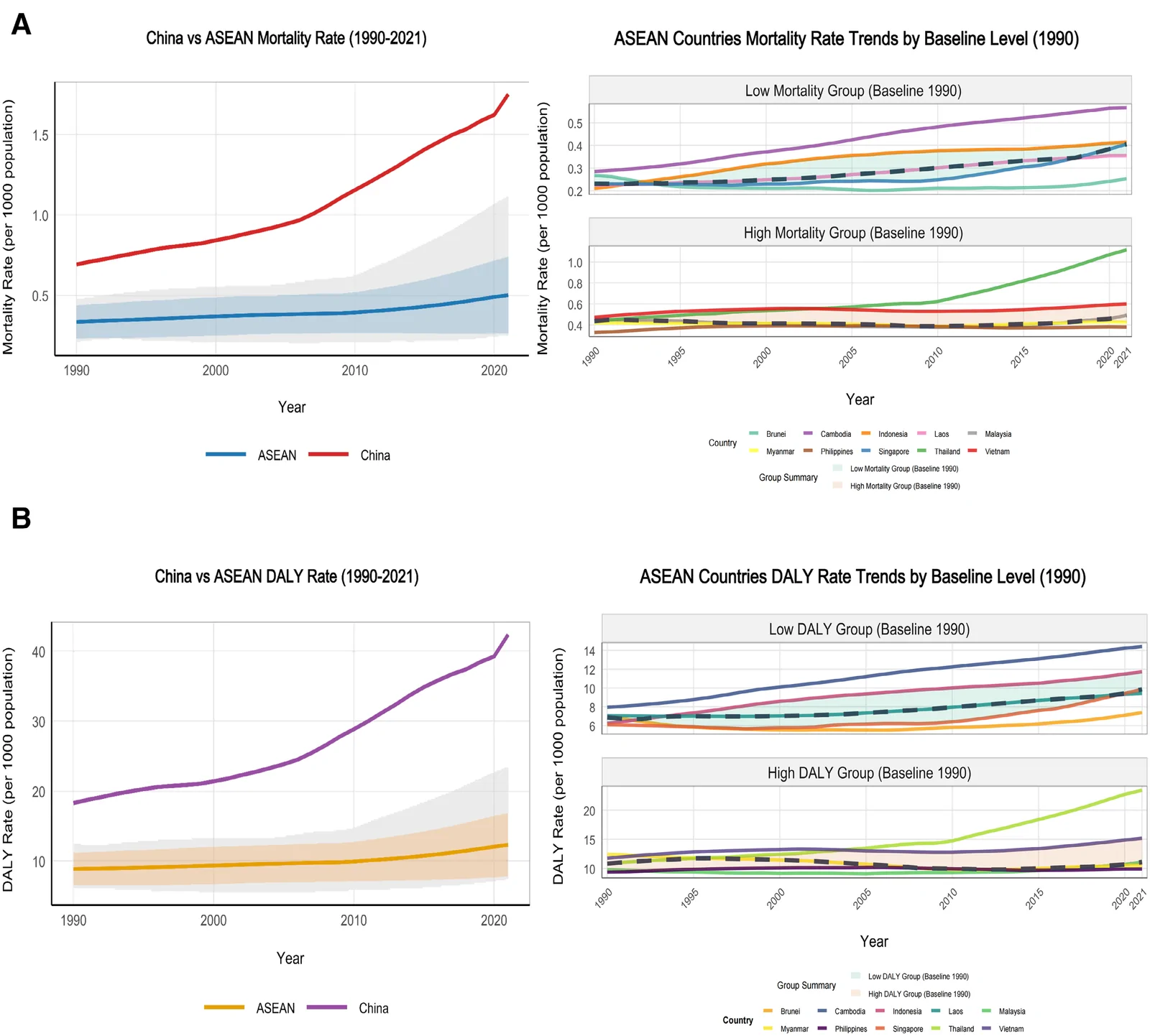
Changes in mortality rate (A) and DALY rate (B) in China and ASEAN countries from 1990 to 2021. (A) Mortality rates (per 100,000 population). Left panel: trends for 2 baseline-defined mortality groups (High and Low, based on 1990 rates), with individual ASEAN countries shown in distinct colors. Right panel: direct comparison between China (red) and the ASEAN average (blue). (B) DALY rates (per 100,000 population), with the same grouping and scheme as in (A). ASEAN = Association of Southeast Asian Nations; DALY = disability-adjusted life year. Data source: Global Burden of Disease 2021.

### 4.2. Analysis of gender differences in the burden of ADOD disease attributed to tobacco

#### 4.2.1. Number of deaths and DALYs

Between 1990 and 2021, both the number of deaths and the DALYs in males with ADOD attributed to tobacco were higher than those in females in China and ASEAN countries, and the mortality rate and DALY rates were also higher than those in females (Table 1).

A pronounced upward trend was observed in both the number of deaths and DALYs among males, particularly in China, where the increase was most pronounced. Similar rapid growth patterns were also documented in other ASEAN countries, such as Indonesia, Vietnam, and Thailand. In contrast, the total number of female deaths and DALYs showed a gradual, relatively moderate upward trend. The overall magnitude of male deaths substantially exceeded that of females, being approximately fivefold higher. Notably, in Vietnam in 2021, the number of male deaths reached approximately 8 times that of females. The total number of male DALYs considerably surpassed that of females. As of 2021, the number of male DALYs in China was about 3 times that of females, and the number in Indonesia was about 7 times that of females. These patterns collectively indicate substantial gender differences (Table 1, Fig. 3A–D).

**Figure 3.**
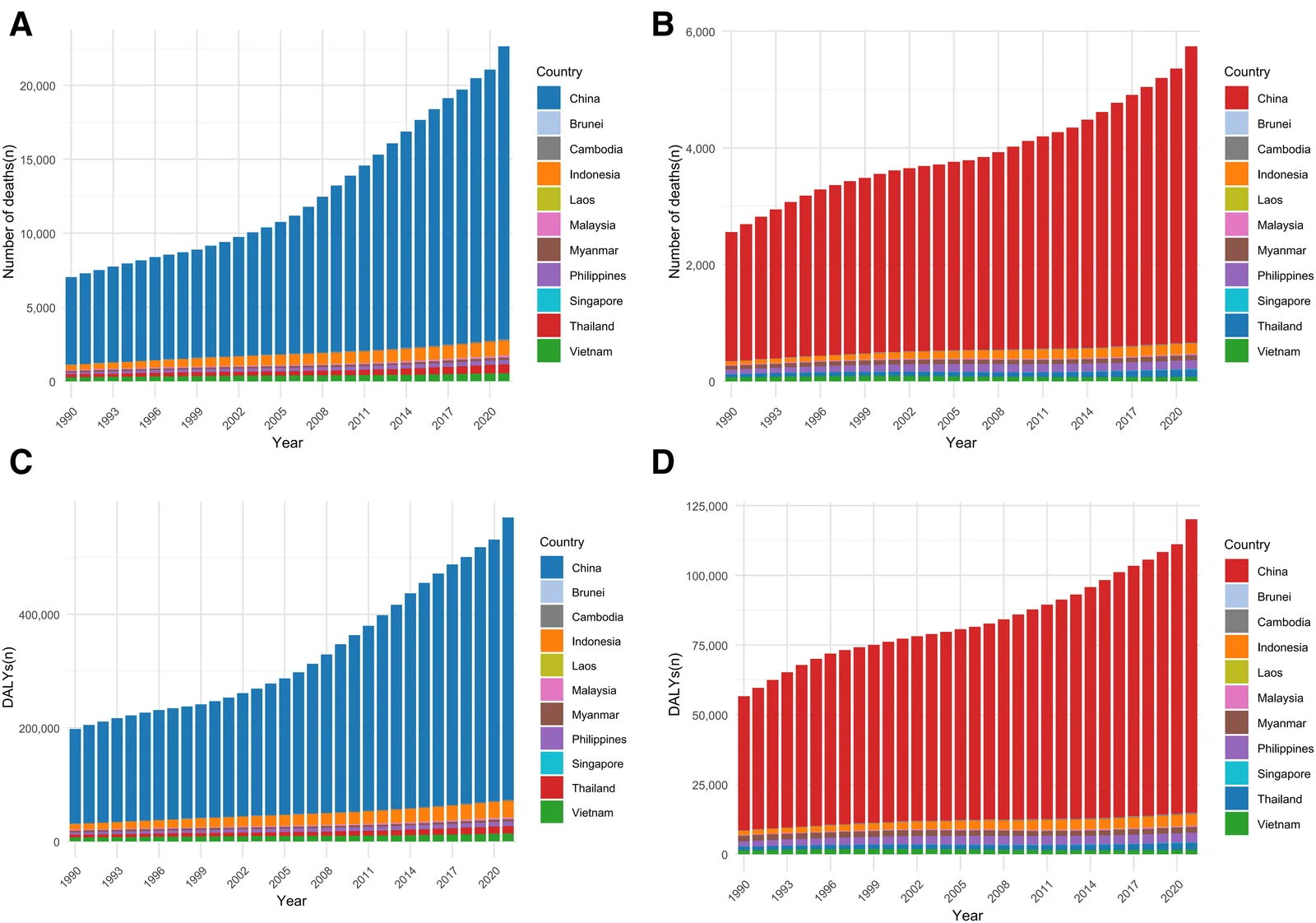
Temporal changes in mortality (A, B) and DALYs (C, D) due to ADOD in China and ASEAN countries, 1990–2021, stratified by sex. Panels A and B present data for males, with China highlighted in blue to facilitate visual distinction given its substantially larger disease burden. Panels C and D present data for females, with China highlighted in red. Mortality counts and DALYs are shown as annual absolute numbers. DALYs = disability-adjusted life years; ADOD = Alzheimer’s disease and other dementias.

### 4.3. Trend analysis of annual percent change (APC) in mortality rate and DALY rates

Overall, from 1990 to 2021, most ADOD mortality attributable to tobacco in China and ASEAN countries showed an upward trend. The annual percentage change (APC) in mortality was positive for China (3.02%), Cambodia (2.38%), Indonesia (2.00%), Laos (1.67%), Singapore (1.67%), Thailand (2.79%), Vietnam (0.38%), and the Philippines (0.21%), all statistically significant (*P* < .001). Among these, China and Thailand exhibited substantial increases (APC > 2.5%), while the Philippines showed a minimal increase (APC = 0.21%). In contrast, Brunei (−0.15%), Malaysia (−0.17%), and Myanmar (−0.04%) had negative APCs close to zero, which were not statistically significant (*P* > .05), indicating stable mortality levels (Table 2).

**Table 2:** Annual Percent Change (APC) in Mortality and DALY Rates (1990–2021)

Country	Mortality APC (95% CI)	*P*	Trend	DALY Rate APC (95% CI)	*P*	Trend
China	3.02 (2.84~3.21)	<.001	Increase	2.75 (2.56~2.94)	<.001	Increase
Brunei	−0.15 (−0.44~0.15)	.311	Stable	0.34 (0.03~0.65)	.035	Increase
Cambodia	2.38 (2.27~2.50)	<.001	Increase	1.99 (1.89~2.08)	<.001	Increase
^Indonesia^	2.00 (1.75~2.28)	<.001	Increase	1.88 (1.72~2.04)	<.001	Increase
Laos	1.67 (1.60~1.74)	<.001	Increase	1.05 (0.91~1.20)	<.001	Increase
Malaysia	−0.17 (−0.45~0.11)	.233	Stable	0.28 (0.12~0.44)	.0015	Increase
Myanmar	−0.04 (−0.15~0.07)	.5	Stable	−0.76 (−0.90~−0.61)	<.001	Decrease
Philippines	0.21 (0.05~0.37)	.012	Increase	0.04 (−0.05~0.13)	.401	Stable
Singapore	1.67 (1.32~2.02)	<.001	Increase	1.54 (1.20~1.87)	<.001	Increase
Thailand	2.79 (2.47~3.10)	<.001	Increase	2.40 (2.15~2.65)	<.001	Increase
Vietnam	0.38 (0.25~0.52)	<.001	Increase	0.45 (0.32~0.59)	<.001	Increase

APC = annual percent change. CI = confidence interval.

*P* < .05 was considered statistically significant for trend classification.

Regarding DALY rates, statistically significant increases were observed in China (2.75%), Cambodia (1.99%), Indonesia (1.88%), Laos (1.05%), Malaysia (0.28%), Singapore (1.54%), Thailand (2.40%), and Vietnam (0.45%), all *P* < .001. Brunei also showed a modest increase (0.34%, *P* < .05). The Philippines had a negligible, non‑significant APC of 0.04% (*P* > .05), suggesting no clear change in disease burden. Myanmar was the only country with a statistically significant decline in DALY rates (APC = −0.76%, *P* < .001) (Table 2).

### 4.4. Correlation analysis of SDI with mortality rate and DALY rates

From 1990 to 2021, a nonlinear association was observed between the SDI and the tobacco-attributable mortality and DALY rates for ADOD in China and ASEAN member states. Overall Spearman rank correlation analysis indicated no significant monotonic correlation between SDI and mortality rates (ρ = 0.049, *P* = .36), whereas a significant but weak monotonic correlation was found between SDI and DALY rates (ρ = −0.139, *P* = .009). Stratified analysis by SDI revealed that when SDI exceeded 0.80, increases in mortality and DALY rates were gradual. Singapore and Brunei, which fall within this SDI range, exhibited relatively low disease burdens. In the SDI range of 0.70 to 0.79, countries such as Thailand and Malaysia showed a rising but relatively moderate trend in mortality and DALY rates. Among countries with an SDI between 0.60 and 0.69 (China, Indonesia, Vietnam, and the Philippines), the disease burden increased more slowly in the latter 3 countries, whereas China experienced a more pronounced increase. At SDI levels below 0.60, Cambodia, Laos, and Myanmar demonstrated a marked upward trend in both mortality and DALY rates. In summary, the relationship between SDI and tobacco-attributable aortic and peripheral arterial disease mortality and DALY rates in China and ASEAN countries is a nonlinear association. Although a significant negative correlation exists between SDI and DALY rate, the strength of this association is weak (ρ = −0.139), and no significant correlation was found for mortality rates. These findings suggest that the association between socioeconomic development level and tobacco-related disease burden may exhibit stage-specific characteristics (Fig. 4A, B).

**Figure 4.**
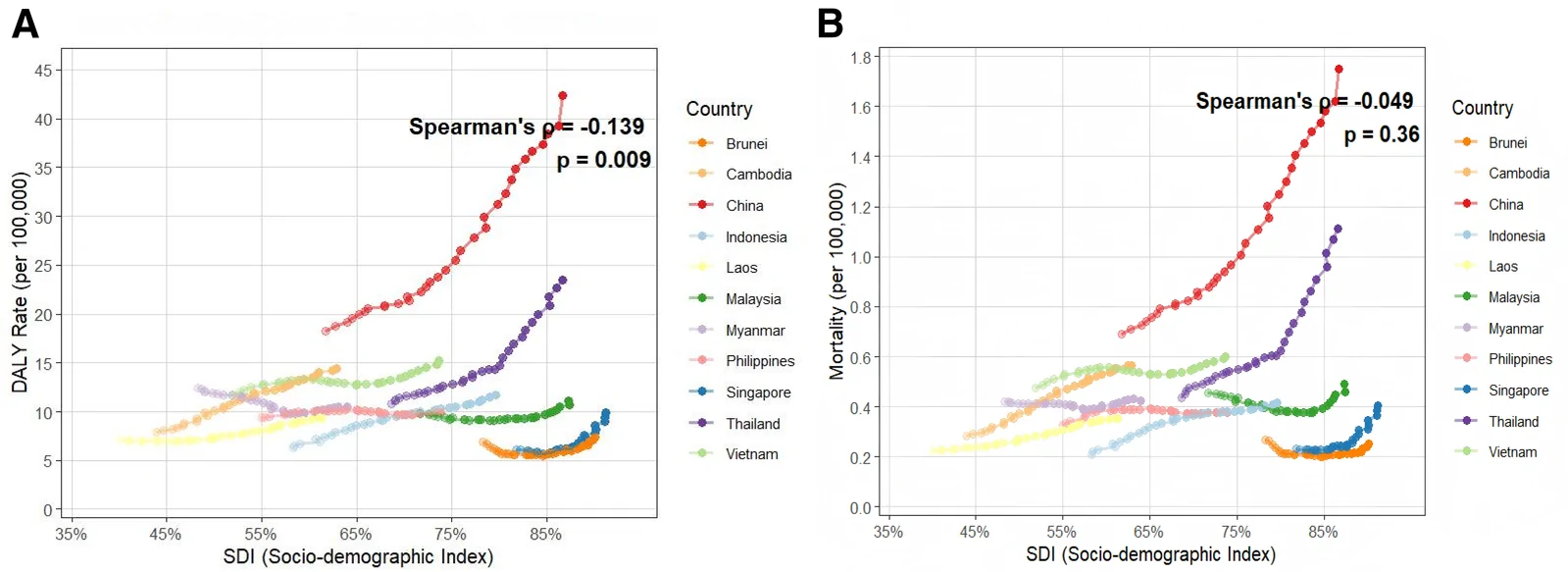
Correlation Analysis between SDI and Mortality (A) and DALY Rates (B). (A) Scatter plot showing the correlation between SDI and mortality rates. Each point represents a country, color-coded by SDI quintile. (B) Scatter plot illustrating the correlation between SDI and DALY rates. SDI = Socio-demographic Index; DALY = disability-adjusted life year.

### 4.5. ARIMA model predicts ADOD mortality rate over the next 10 years

For all countries, ARIMA models were automatically selected using auto.arima with p, d, q ≤ 3 based on minimum AICc. The most frequent models were ARIMA(0,1,1) and ARIMA(1,1,1), and no country required second-order differencing. Using China as an example, the final model was ARIMA(1,1,1) (AICc = 123.4). During the training period (1990–2021), the median RMSE across countries was 0.13 per 100,000, and the median MAPE was 8.1%. Residual diagnostics confirmed that all models passed the Ljung–Box test (*P* > .05), indicating no significant residual autocorrelation.

The ARIMA model projections indicate that mortality rate trends in China and ASEAN countries from 2022 to 2035 can be categorized into 5 distinct patterns: rising (Cambodia, Laos, Thailand, Indonesia, China, Vietnam, Singapore), declining (Philippines), rising-then-falling (Brunei), falling-then-rising (Myanmar), and stable (Malaysia). Among the countries with a rising mortality rate, China exhibits the most pronounced increase, rising from 1.87 to 3.53 per 100,000 between 2022 and 2035. It is followed by Thailand, with an increase from 1.15 to 1.75 per 100,000. Vietnam displays the most gradual upward trend, edging up only from 0.60 to 0.63 per 100,000; however, its wide confidence interval indicates greater uncertainty in this projection. The Philippines is the only country projected to experience a decline, from 0.37 to 0.35 per 100,000. However, as its prediction interval encompasses both an increase and a decrease, an upward trend in the later period remains possible. Between 2022 and 2035, Brunei exhibits a rising-then-falling pattern, increasing from 0.26 to 0.27 per 100,000 during 2022 to 2027 before declining to 0.22 by 2035. Conversely, Myanmar demonstrates a falling-then-rising pattern. Its rate is projected to decrease from 0.42 to 0.40 per 100,000 between 2022 and 2027, followed by an increase to 0.41 by 2035, resulting in a minor net change. The projections for Malaysia indicate a stable mortality rate over the next 15 years, fluctuating slightly around the historical average of 0.41 per 100,000. In summary, despite the diversity in trends, the majority of countries are projected to exhibit an upward trajectory during the 2022–2035 period (Fig. 5).

**Figure 5.**
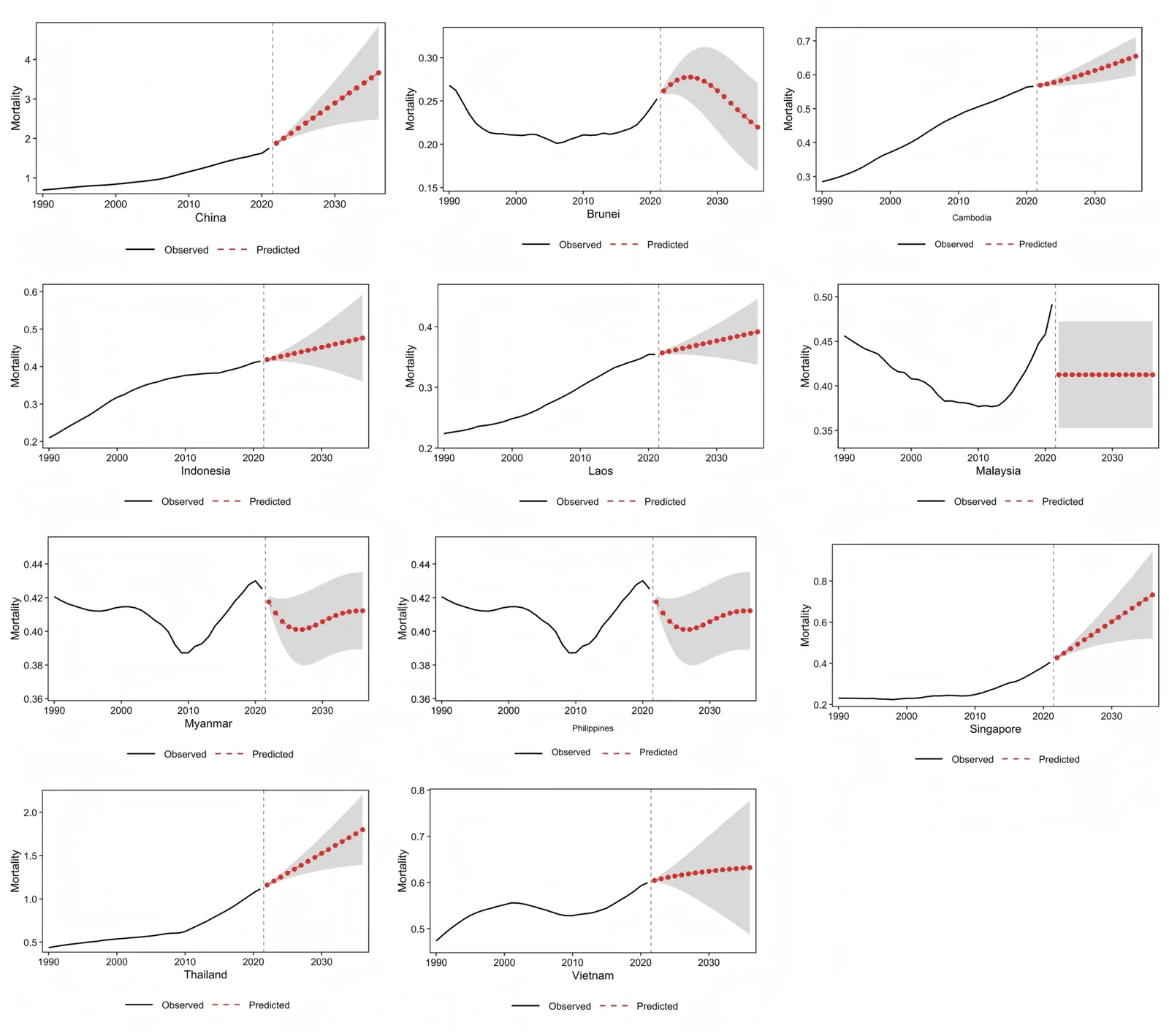
Mortality Rate Projections for China and ASEAN Countries (2022–2035).Solid lines represent observed mortality rates from 1990 to 2021; dashed lines represent predicted mortality rates from 2022 to 2035. ASEAN = Association of Southeast Asian Nations.

## 5. Discussion

Numerous epidemiological and basic studies have demonstrated that tobacco exposure – including active smoking, secondhand smoke inhalation, and smokeless tobacco use – can significantly increase the risk of ADOD through multiple potential mechanisms.^[6,14]^ Experimental evidence suggests that nicotine, nitrosamines, and oxidative substances present in tobacco can induce chronic neuroinflammation in the central nervous system and are associated with pathological processes such as β-amyloid deposition and tau protein hyperphosphorylation.^[15]^ Furthermore, secondhand smoke exposure is believed to cause vascular endothelial dysfunction and cerebral hypoperfusion, whereas heavy metals and nitrosamines found in smokeless tobacco products can be absorbed directly into the circulatory system through the oral mucosa, thereby inducing systemic oxidative stress.^[16]^ Collectively, these pathways provide a potential biological explanation for how tobacco exposure accelerates cognitive decline.

The present study found that, between 1990 and 2021, the overall burden of tobacco-attributable ADOD exhibited an upward trend in both China and the ten ASEAN countries, albeit with marked differences across nations. China’s disease burden was significantly higher than that of the other ASEAN countries, a disparity reflected not only in absolute numbers but also in the growth trajectory. From an ecological perspective, this disparity shows consistency at the population level with the following contextual factors: first, China has maintained a persistently high smoking prevalence, creating a sustained and extensive foundation for risk exposure.^[2]^ Second, the combination of a large population base and accelerating population aging has further amplified the overall magnitude of tobacco-related health damage. In contrast, most ASEAN countries present distinct characteristics in terms of tobacco control policies, population structure, and smoking prevalence levels, which together may contribute to the regional pattern of disparity. As an ecological study, this analysis is based on country-level aggregate data and cannot establish causal relationships between tobacco exposure and ADOD at the individual level. Therefore, the following discussion of cross-country differences is intended to describe consistency between the observed patterns and potential contextual factors and to generate hypotheses for future research, rather than to draw causal inferences.

Among the ASEAN countries, Thailand bears the most prominent burden of tobacco-attributable ADOD. Between 1990 and 2021, both its mortality rate and DALY rates showed a significant upward trend, with the magnitude of increase ranking second only to that of China.^[17]^ Observed data indicate that this upward trend coincides temporally with Thailand’s accelerating population aging and persistently high levels of tobacco exposure over the same period. However, because the ecological design of this study cannot distinguish whether this increase originates from the cumulative risk of past smoking or from improvements in diagnostic capacity, no causal inference can be drawn.

In contrast to Thailand, GBD 2021 data indicate that the burden of tobacco-attributable ADOD in the Philippines has grown relatively slowly, while the country’s adult smoking prevalence remains at a relatively high level and the implementation of tobacco control policies has been comparatively slow.^[1]^ Furthermore, due to political unrest in some ASEAN countries (e.g., Myanmar), data collection has been interrupted since 2015. The GBD Study employed model extrapolation for these countries, resulting in mortality trend estimates (e.g., an APC of −0.04% for Myanmar) that are less reliable than those for other countries.^[17]^

In contrast, Brunei Darussalam exhibits a relatively low burden of tobacco-attributable ADOD. Observations indicate that the country has established a relatively stringent tobacco control framework, including ratification of the WHO Framework Convention on Tobacco Control, enactment of the Tobacco Order, and the implementation of smoking bans during Ramadan. These measures coexist temporally with the low disease burden; however, ecological data cannot confirm a causal link between them.

The disease burden of ADOD in Malaysia decreased to its lowest point around 2010 and has shown an upward trend thereafter. This temporal pattern invites several possible explanations, although causal inference is not possible with the present ecological data. The country implemented tobacco product control regulations relatively early in 2004, with potential population-level health effects possibly emerging several years later (around 2010); the increase after 2010 coincides with the aging phase of long-term cumulative risks among Malaysian men who experienced high smoking prevalence during the 1970s to 1980s.^[5,17]^ Furthermore, improvements in diagnostic capacity resulting from economic development and medical technological advances may also partially explain the observed increase in burden after 2010. It should be emphasized that the associations among the above factors remain to be validated by individual-level studies.

Previous studies have suggested a negative correlation between the Socio-demographic Index (SDI) and the burden of smoking-attributable ADOD.^[18]^ The present study further observed that the relationship between these 2 variables is not simply linear but rather exhibits stage-specific characteristics. High-SDI countries (e.g., Singapore, Brunei) have generally implemented comprehensive smoke-free legislation in public places and high tobacco taxes, whereas low-SDI countries (e.g., Cambodia, Laos, Myanmar) lack national smoking bans. The relatively lower burden observed in some low-SDI countries may be related to their smaller population sizes and incomplete data reporting. Of note, although China is a middle-SDI country, its tobacco-attributable disease burden is substantially higher than that of ASEAN countries, which is consistent with contextual factors such as China’s large smoking population, uneven regional development, and rapid population aging.^[17,19,20]^

It is generally accepted that women have a higher biological susceptibility to Alzheimer’s disease (AD), resulting in a disease burden far greater than that of men.^[2,21]^ In the present study, however, the burden of tobacco-attributable ADOD is substantially higher in males than in females across both China and the ASEAN countries, which is consistent with the global pattern where male smoking rates far exceed female smoking rates (by approximately fivefold).^[22]^ Taking China as an example, the smoking rate among males has remained persistently high, resulting in approximately 4 times higher ADOD deaths and DALY rates in males compared to females. Nevertheless, a noteworthy observation is that since approximately 2020, the annual growth rate of ADOD-related DALYs among Chinese females has accelerated markedly, substantially narrowing the gender gap in DALY increases. From an ecological perspective, this change shows temporal consistency with the following contextual characteristics: an intergenerational increase in active smoking rates among younger and rural females^[17]^; long-term betel nut chewing containing tobacco among females in certain regions^[23]^; and the cumulative effects of long-term secondhand smoke exposure among females.^[24]^ However, given the ecological nature of our data, whether these factors have directly contributed to the observed trend remains a hypothesis that warrants further investigation. In most ASEAN countries, the burden of tobacco-attributable ADOD is also higher among males than among females. In 2021, the number of tobacco-attributable ADOD deaths among Vietnamese males was approximately 8 times that among females, and the DALY rate among Indonesian males was approximately 7 times that among females. However, in countries such as the Philippines, Indonesia, and Cambodia, the annual mortality growth rate is higher among females than among males. Previous studies have documented certain contextual characteristics in these countries that could offer possible explanations for this observation: Cambodian females commonly use betel nut products containing tobacco, with their salivary cotinine levels comparable to those of male smokers; while females in the Philippines and Indonesia have high levels of long-term secondhand smoke exposure,^[25]^Nevertheless, we acknowledge that these are potential correlates rather than confirmed causal factors, and this interpretation remains speculative pending further analytical studies.

Overall, as an ecological and observational study, our findings indicate associations at the population level rather than causal relationships. Although China and ASEAN countries have achieved notable progress in economic integration, with bilateral trade reaching 6.41 trillion RMB (approximately 878.2 billion USD) in 2021, making ASEAN China’s largest trading partner for consecutive years,^[26]^ cooperation in health-related areas, particularly tobacco control and the prevention of Alzheimer’s disease and other dementias, has lagged behind.^[27]^ Given the inherent uncertainties in long-term projections of disease burden, any policy implications should be considered preliminary and exploratory. With this caveat, potential avenues for future collaboration between China and ASEAN countries may tentatively include: establishing a cross-border platform for joint disease surveillance and response, creating a platform for sharing legislative experiences on tobacco control, and enhancing technical exchanges on early screening for ADOD.

The limitations of this study are primarily reflected in the following 3 aspects. First, the data were derived from the GBD 2021 database. Although this database is highly systematic, differences in data quality across countries with varying levels of development may introduce bias. Second, due to constraints inherent to the database, the study was unable to control for individual-level factors (e.g., education, lifestyle) or unmeasured confounders. Furthermore, the calculation of attributable fractions relies on estimates of exposure levels and relative risks, which may introduce information bias. Future studies incorporating original research are needed to further validate these findings. Third, the disease burden of ADOD is determined by multiple factors, including aging, genetics, cardiovascular and cerebrovascular diseases, unhealthy lifestyles, and hearing impairment, among which tobacco use is only one contributing factor. In addition, as an ecological study, the observed associations at the population level cannot be directly inferred as causal relationships at the individual level, posing a potential risk of ecological fallacy. Therefore, when interpreting the conclusions regarding tobacco use in this study, caution should be exercised, and the findings should be considered within the broader context of multifactorial etiology.

Note: The GBD Study adheres to the standards and definitions of the WHO in terms of geographic regional division, so as to maintain neutrality and avoid disputes over territorial claims. Therefore, the descriptions of any country, territory, city, or region, as well as statements related to their borders and sovereignty, in this paper shall not be construed as representing the official positions of the research team, its affiliated institutions, or the funders.

## Acknowledgments

The authors sincerely thank the Institute for Health Metrics and Evaluation (IHME) and the broader Global Burden of Disease collaborative for producing and making the GBD 2021 data publicly available. We also thank our colleagues at the Faculty of Zhuang Clinical Medicine for their valuable feedback during the internal review of this study.

## Author contributions

**Conceptualization:** Piaoyun Wen, Wenwen Liu, Yanjiang Li.

**Data curation:** Piaoyun Wen, Wenwen Liu, Yanjiang Li.

**Formal analysis:** Piaoyun Wen, Wenwen Liu, Yanjiang Li, Mengmeng Liao, Haofeng Qin, Jiaxi Ye, Ziyong Deng, Shuqi Cui, Yongli Zhao, Xiaowen Wang, Yingzi Huang, Hui Qin.

**Investigation:** Piaoyun Wen, Wenwen Liu, Yanjiang Li, Mengmeng Liao, Haofeng Qin, Jiaxi Ye, Ziyong Deng, Shuqi Cui, Yongli Zhao, Xiaowen Wang, Yingzi Huang, Hui Qin.

**Methodology:** Piaoyun Wen, Wenwen Liu, Yanjiang Li.

**Resources:** Huamin Tang, Kaihua Wang, Qijing Qin.

**Supervision:** Huamin Tang, Kaihua Wang, Qijing Qin.

**Validation:** Piaoyun Wen, Wenwen Liu, Yanjiang Li, Mengmeng Liao, Haofeng Qin, Jiaxi Ye, Ziyong Deng, Shuqi Cui, Yongli Zhao, Xiaowen Wang, Yingzi Huang, Hui Qin.

Visualization: Piaoyun Wen.

**Funding acquisition:** Huamin Tang, Kaihua Wang, Qijing Qin.

**Project administration:** Huamin Tang, Kaihua Wang, Qijing Qin.

**Writing – original draft:** Piaoyun Wen, Wenwen Liu, Yanjiang Li.

**Writing – review & editing:** Piaoyun Wen, Wenwen Liu, Yanjiang Li, Mengmeng Liao, Haofeng Qin, Jiaxi Ye, Ziyong Deng, Shuqi Cui, Yongli Zhao, Xiaowen Wang, Yingzi Huang, Hui Qin.

## References

[1] Steinmetz JD Seeher KM Schiess N. Global, regional, and national burden of disorders affecting the nervous system, 1990–2021: a systematic analysis for the Global Burden of Disease Study 2021. Lancet Neurol. 2024;23:344–81. 10.1016/S1474-4422(24)00038-3 PMC10949203 38493795

[2] Nichols E Steinmetz JD Vollset SE. Estimation of the global prevalence of dementia in 2019 and forecasted prevalence in 2050: an analysis for the Global Burden of Disease Study 2019. Lancet Public Health. 2022;7:e105–25. 10.1016/S2468-2667(21)00249-8 PMC8810394 34998485

[3] Alzheimer’s Disease International. World Alzheimer report 2023: reducing dementia risk: never too early, never too late. 2023. https://www.alzint.org/resource/world-alzheimer-report-2023/. Accessed November 27, 2025.

[4] Murray CJL Aravkin AY Zheng P. Global burden of 87 risk factors in 204 countries and territories, 1990–2019: a systematic analysis for the Global Burden of Disease Study 2019. Lancet. 2020;396:1223–49. 10.1016/S0140-6736(20)30752-2 PMC7566194 33069327

[5] Durazzo TC Mattsson N Weiner MW; Alzheimer’s Disease Neuroimaging Initiative. Smoking and increased Alzheimer’s disease risk: a review of potential mechanisms. Alzheimers Dement. 2014;10(3 suppl):S122–45. 10.1016/j.jalz.2014.04.009 PMC4098701 24924665

[6] Livingston G Huntley J Sommerlad A. Dementia prevention, intervention, and care: 2020 report of the Lancet Commission. Lancet. 2020;396:413–46. 10.1016/S0140-6736(20)30367-6 PMC7392084 32738937

[7] Kaur J Rinkoo A Gouda H Prasad V Pendse R. Implementation of MPOWER package in the South-East Asia region: evidence from the WHO report on the global tobacco epidemic (2009-2021). Asian Pac J Cancer Prev. 2021;22(suppl 2):71–80. 10.31557/APJCP.2021.22.S2.71 34780141

[8] Yu C Chen J. Global Burden of substance use disorders among adolescents during 1990-2021 and a forecast for 2022-2030: an analysis for the Global Burden of Disease 2021. BMC Public Health. 2025;25:1012. 10.1186/s12889-025-22107-6 PMC11909827 40087641

[9] Brauer M Roth GA Aravkin AY. Global burden and strength of evidence for 88 risk factors in 204 countries and 811 subnational locations, 1990–2021: a systematic analysis for the Global Burden of Disease Study 2021. Lancet. 2024;403:2162–203. 10.1016/S0140-6736(24)00933-4 PMC11120204 38762324

[10] Vos T Lim SS Abbafati C. Global burden of 369 diseases and injuries in 204 countries and territories, 1990–2019: a systematic analysis for the Global Burden of Disease Study 2019. Lancet. 2020;396:1204–22. 10.1016/S0140-6736(20)30925-9 PMC7567026 33069326

[11] Kim HJ Fay MP Feuer EJ Midthune DN. Permutation tests for joinpoint regression with applications to cancer rates. Stat Med. 2000;19:335–51. 10.1002/(sici)1097-0258(20000215)19:3<335::aid-sim336>3.0.co;2-z 10649300

[12] Box GEP Jenkins GM Reinsel GC Ljung GM. Time Series Analysis: Forecasting and Control. 5th ed. Wiley; 2015.

[13] Chen R Wei J. Reversed gender burden of pancreatic cancer attributable to high BMI: a GBD 2021 analysis of 204 countries with projections to 2041. Cancer Control. 2025;32:10732748251372674. 10.1177/10732748251372674 PMC12399818 40884075

[14] Deal JA Power MC Palta P. Relationship of cigarette smoking and time of quitting with incident dementia and cognitive decline. J Am Geriatr Soc. 2020;68:337–45. 10.1111/jgs.16228 PMC7002272 31675113

[15] Kinney JW Bemiller SM Murtishaw AS Leisgang AM Salazar AM Lamb BT. Inflammation as a central mechanism in Alzheimer’s disease. Alzheimers Dement (NY). 2018;4:575–90. 10.1016/j.trci.2018.06.014 PMC6214864 30406177

[16] Debette S Markus HS. The clinical importance of white matter hyperintensities on brain magnetic resonance imaging: systematic review and meta-analysis. BMJ. 2010;341:c3666. 10.1136/bmj.c3666 PMC2910261 20660506

[17] Reitsma MB Kendrick PJ Ababneh E. Spatial, temporal, and demographic patterns in prevalence of smoking tobacco use and attributable disease burden in 204 countries and territories, 1990–2019: a systematic analysis from the Global Burden of Disease Study 2019. Lancet. 2021;397:2337–60. 10.1016/S0140-6736(21)01169-7 PMC8223261 34051883

[18] Naghavi M Ong KL Aali A. Global burden of 288 causes of death and life expectancy decomposition in 204 countries and territories and 811 subnational locations, 1990–2021: a systematic analysis for the Global Burden of Disease Study 2021. Lancet. 2024;403:2100–32. 10.1016/S0140-6736(24)00367-2 PMC11126520 38582094

[19] He Z Zhang H Hu G. The current status, trends, and challenges of Alzheimer’s disease and other dementias in Asia (1990–2036). Front Public Health. 2025;13:1583339. 10.3389/fpubh.2025.1583339 PMC12185285 40556904

[20] Fu H Huang H Liao S Dai Z. Global burden of Alzheimer’s disease and other dementias (1990–2021): inequality, frontier, and decomposition analysis. Front Aging Neurosci. 2025;17:1637029. 10.3389/fnagi.2025.1637029 PMC12489941 41049534

[21] World Health Organization. WHO report on the global tobacco epidemic 2021: addressing new and emerging products. https://www.who.int/publications/i/item/9789240032095. Accessed November 28, 2025.

[22] World Health Organization. WHO global report on trends in prevalence of tobacco use 2000–2030. 2024. https://www.who.int/publications/i/item/9789240039322. Accessed October 12, 2025.

[23] Liu L Yu J Li L. Alpha7 nicotinic acetylcholine receptor is required for amyloid pathology in brain endothelial cells induced by Glycoprotein 120, methamphetamine and nicotine. Sci Rep. 2017;7:40467. 10.1038/srep40467 PMC5225415 28074940

[24] Serrano-Pozo A Das S Hyman BT. APOE and Alzheimer’s disease: advances in genetics, pathophysiology, and therapeutic approaches. Lancet Neurol. 2021;20:68–80. 10.1016/S1474-4422(20)30412-9 PMC8096522 33340485

[25] WHO Global Report on Trends in Prevalence of Tobacco Use 2000-2025. World Health Organization; 2021.

[26] General Administration of Customs of the People’s Republic of China. http://english.customs.gov.cn/. Accessed November 29, 2025.

[27] World Health Organization. For the future: towards the healthiest and safest region. https://www.who.int/publications/i/item/WPR-2020-RDO-001. Accessed October 15, 2025.

